# Supplementary material for: Systematic review and meta-analysis of the effectiveness and perinatal outcomes of COVID-19 vaccination in pregnancy
Source: Nat Commun. 2022 May 10;13:2414. doi: 10.1038/s41467-022-30052-w (PMC9090726; doi:10.1038/s41467-022-30052-w)
Supplement: Supplementary file 1 — Supplementary Information [file 41467_2022_30052_MOESM1_ESM.pdf]

SUPPLEMENTARY INFORMATION

Title: Systematic Review and meta-analysis of the effectiveness and Perinatal Outcomes of COVID-19 Vaccination in Pregnancy

Smriti Prasad, MRCOG [1], Erkan Kalafat, MD MSc [2,3], Helena Blakeway, MBChB [1], Rosemary Townsend, MRCOG[4,5], Pat O'Brien, FRCOG[6,7], Edward Morris, FRCOG[6,8], Tim Draycott, FRCOG[6,9], Shakila Thangaratinam, FRCOG[10], Kirsty Le Doare, PhD[11], Shamez Ladhani, PhD[12,13], Peter von Dadelszen, FRCOG[14], Laura A. Magee, FRCOG [14], Paul Heath, FRCPCH[11], Asma Khalil, MRCOG[1,5*]

[1]Fetal Medicine Unit, St George's Hospital, St George's University of London, UK

[2]Middle East Technical University, Faculty of Arts and Sciences, Department of Statistics, Ankara, Turkey

[3]Koc University, School of Medicine, Department of Obstetrics and Gynaecology, Istanbul, Turkey

[4]Usher Institute of Population Health Sciences and Informatics, University of Edinburgh, Edinburgh, UK

[5]Vascular Biology Research Centre, Molecular and Clinical Sciences Research Institute, St George's University of London, UK

[6]The Royal College of Obstetricians and Gynaecologists, London, UK

[7]University College London Hospitals NHS Foundation Trust, London, UK

[8]Norfolk and Norwich University Hospitals NHS Foundation Trust, Norwich, Norfolk, UK

[9]North Bristol NHS Trust Department of Women's Health, Westbury on Trym, UK

[10]Institute of Metabolism and Systems Research, WHO Collaborating Centre for Women's Health, University of Birmingham, Birmingham, UK

[11]Paediatric Infectious Diseases Research Group and Vaccine Institute, Institute of Infection and Immunity, St George's University of London, London, UK

[12] Immunisation and Countermeasures Division, Public Health England

[13]British Paediatric Surveillance Unit, Royal College of Paediatrics and Child Health

[14]Institute of Women and Children's Health, School of Life Course Sciences, King's College London, London, UK

Contents **Page**

Table S1. A list of the excluded studies with reasons for their exclusion……………………3-6
Table S2. Characteristics of the studies included in the systematic review………………..7-18
Table S3. ROBINS-I risk of bias assessment of observational studies………………........... 19
Table S4. Cochrane Risk of Bias Tool 2 for randomized trials……………………………….. 20
Table S5. Literature search strategy. 21

Table S1. A list of the excluded studies with reasons for their exclusion.

	**Studies**	**Exclusion reason**
1.	Rottenstreich A, Zarbiv G, Oiknine-Djian E, Zigron R, Wolf DG, Porat S. Efficient maternofetal transplacental transfer of anti- SARS-CoV-2 spike antibodies after antenatal SARS-CoV-2 BNT162b2 mRNA vaccination. Clin Infect Dis. 2021 Apr 3;ciab266.	Unsuitable outcomes (transplacental transfer of antibodies)
2.	Burd I, Kino T, Segars J. The Israeli study of Pfizer BNT162b2 vaccine in pregnancy: considering maternal and neonatal benefits. J Clin Invest. 2021 Jul 1;131(13):150790.	Unsuitable study design (review commentary)
3.	Riviello C, Pontello V. Maternal and neonatal SARS-CoV-2 antibodies assessment after mRNA maternal vaccination in the third trimester of pregnancy. Int J Gynaecol Obstet. 2021 Sep;154(3):565–6..	Unsuitable study design (case report)
4.	Paul G, Chad R. Newborn antibodies to SARS-CoV-2 detected in cord blood after maternal vaccination – a case report. BMC Pediatrics. 2021 Mar 22;21(1):138.	Unsuitable study design (case report)
5.	Male V. Are COVID-19 vaccines safe in pregnancy? Nat Rev Immunol. 2021 Apr;21(4):200–1.	Unsuitable study design (comment)
6.	Atyeo C, DeRiso EA, Davis C, et al. COVID-19 mRNA vaccines drive differential Fc-functional profiles in pregnant, lactating, and non-pregnant women. Preprint. *bioRxiv*. 2021;2021.04.04.438404. Published 2021 Apr 5. doi:10.1101/2021.04.04.438404	Unsuitable outcomes (humoral vaccine response)
7.	Orvieto R, Noach-Hirsh M, Segev-Zahav A, Haas J, Nahum R, Aizer A. Does mRNA SARS-CoV-2 vaccine influence patients' performance during IVF-ET cycle? Reprod Biol Endocrinol. 2021 May 13;19:69.	Unsuitable outcomes (stimulation characteristics and embryological variables in IVF cycle)
8.	Ciapponi A, Bardach A, Mazzoni A, Alconada T, Anderson SA, Argento FJ, et al. Safety of components and platforms of COVID-19 vaccines considered for use in pregnancy: A rapid review. Vaccine. 2021 Aug 13;S0264-410X(21)01067-7.	Unsuitable intervention (different vaccine platforms used in pregnant women)

9.	Mose A, Yeshaneh A. COVID-19 Vaccine Acceptance and Its Associated Factors Among Pregnant Women Attending Antenatal Care Clinic in Southwest Ethiopia: Institutional-Based Cross-Sectional Study. Int J Gen Med. 2021;14:2385–95.	Unsuitable outcomes (COVID-19 vaccine acceptance)
10.	Hadar E, Dollinger S, Wiznitzer A. Coronavirus disease and vaccination during pregnancy and childbirth: a review of the Israeli perspective and experience. J Matern Fetal Neonatal Med. 2021 Jun 15;1–12.	Unsuitable study design (review)
11.	Collier A-RY, McMahan K, Yu J, Tostanoski LH, Aguayo R, Ansel J, et al. Immunogenicity of COVID-19 mRNA Vaccines in Pregnant and Lactating Women. JAMA. 2021 Jun 15;325(23):2370–80.	Unsuitable outcomes (antibody and site-specific T cell response)
12.	Razzaghi H, Meghani M, Pingali C, Crane B, Naleway A, Weintraub E, et al. COVID-19 Vaccination Coverage Among Pregnant Women During Pregnancy - Eight Integrated Health Care Organizations, United States, December 14, 2020-May 8, 2021. MMWR Morb Mortal Wkly Rep. 2021 Jun 18;70(24):895–9.	Unsuitable outcomes (vaccine coverage)
13.	Zdanowski W, Waśniewski T. Evaluation of SARS-CoV-2 Spike Protein Antibody Titers in Cord Blood after COVID-19 Vaccination during Pregnancy in Polish Healthcare Workers: Preliminary Results. Vaccines (Basel). 2021 Jun 19;9(6):675.	Unsuitable outcomes (antibody titres in cord blood)
14.	Parums DV. Editorial: Maternal SARS-CoV-2 Infection and Pregnancy Outcomes from Current Global Study Data. Med Sci Monit. 2021 Jul 5;27:e933831.	Unsuitable study design (editorial)<br>Unsuitable intervention (maternal SARS-CoV-2 infection)
15.	Shanes ED, Otero S, Mithal LB, Mupanomunda CA, Miller ES, Goldstein JA. Severe Acute Respiratory Syndrome Coronavirus 2 (SARS-CoV-2) Vaccination in Pregnancy: Measures of Immunity and Placental Histopathology. Obstet Gynecol. 2021 Aug 1;138(2):281–3.	Unsuitable outcomes (antibody response and placental histopathology)
16.	Successful vertical transmission of SARS-CoV-2 antibodies after maternal vaccination - Mehaffey - - Birth - Wiley Online Library [Internet]. [cited 2021 Sep 19]. Available from: https://onlinelibrary.wiley.com/doi/full/10.1111/birt.12582	Unsuitable study design (case report)

17.	Charepe N, Gonçalves J, Juliano AM, et al. COVID-19 mRNA vaccine and antibody response in lactating women: a prospective cohort study. *BMC Pregnancy Childbirth*. 2021;21(1):632. Published 2021 Sep 17. doi:10.1186/s12884-021-04051-6	Unsuitable population (lactating women)
18.	Nana M, Nelson-Piercy C. COVID-19 in pregnancy. *Clin Med (Lond)*. 2021;21(5):e446-e450. doi:10.7861/clinmed.2021-0503	Unsuitable study design (review article)
19.	Duarte G, Coutinho CM, Rolnik DL, et al. Perspectives on administration of COVID-19 vaccine to pregnant and lactating women: a challenge for low- and middle-income countries. *AJOG Glob Rep*. 2021;1(4):100020. doi:10.1016/j.xagr.2021.100020	Unsuitable study design (clinical opinion)
20.	Girardi G, Bremer AA. Scientific Evidence Supporting Coronavirus Disease 2019 (COVID-19) Vaccine Efficacy and Safety in People Planning to Conceive or Who Are Pregnant or Lactating. *Obstet Gynecol*. 2022;139(1):3-8. doi:10.1097/AOG.0000000000004636	Unsuitable study design (current commentary)
21.	Joubert E, Kekeh AC, Amin CN. COVID-19 and novel mRNA vaccines in pregnancy: an updated literature review [published online ahead of print, 2021 Oct 15]. *BJOG*. 2021;10.1111/1471-0528.16973. doi:10.1111/1471-0528.16973	Unsuitable study design (review article)
22.	Moro PL, Panagiotakopoulos L, Oduyebo T, Olson CK, Myers T. Monitoring the safety of COVID-19 vaccines in pregnancy in the US [published online ahead of print, 2021 Nov 10]. *Hum Vaccin Immunother*. 2021;1-9. doi:10.1080/21645515.2021.1984132	Unsuitable study design (review article)
23.	Mangat C, Milosavljevic N. BNT162b2 Vaccination during Pregnancy Protects Both the Mother and Infant: Anti-SARS-CoV-2 S Antibodies Persistently Positive in an Infant at 6 Months of Age. *Case Rep Pediatr*. 2021;2021:6901131. Published 2021 Oct 12. doi:10.1155/2021/6901131	Unsuitable study design (case report)
24.	Sarwal Y, Sarwal T, Sarwal R. Vaccination of pregnant women against COVID-19 in India and Indonesia: Moving beyond the opt-in to the opt-out option. *Int J Gynaecol Obstet*. 2021;155(3):549-550. doi:10.1002/ijgo.13930	Unsuitable study design (opinion paper)
25.	Shook LL, Fallah PN, Silberman JN, Edlow AG. COVID-19 Vaccination in Pregnancy and Lactation: Current Research and Gaps in Understanding. *Front Cell Infect Microbiol*. 2021;11:735394. Published 2021 Sep 16. doi:10.3389/fcimb.2021.735394	Unsuitable study design (review article)

26.	Klein AZ, O'Connor K, Gonzalez-Hernandez G. Toward Using Twitter Data to Monitor Covid-19 Vaccine Safety in Pregnancy: A Proof-of-Concept Study of Cohort Identification [published online ahead of print, 2021 Nov 22]. *JMIR Form Res*. 2021;10.2196/33792. doi:10.2196/33792	Unsuitable outcomes (user reported vaccine uptake)
27.	Karrow NA, Shandilya UK, Pelech S, et al. Maternal COVID-19 Vaccination and Its Potential Impact on Fetal and Neonatal Development. *Vaccines (Basel)*. 2021;9(11):1351. Published 2021 Nov 18. doi:10.3390/vaccines9111351	Unsuitable study design (review article)
28.	Jorgensen SCJ, Burry L, Tabbara N. Role of maternal COVID-19 vaccination in providing immunological protection to the newborn [published online ahead of print, 2021 Nov 23]. *Pharmacotherapy*. 2021;10.1002/phar.2649. doi:10.1002/phar.2649	Unsuitable study design (review article)
29.	Shimabukuro TT, Kim SY, Myers TR, et al. Preliminary Findings of mRNA Covid-19 Vaccine Safety in Pregnant Persons. New England Journal of Medicine. 2021/06/17 2021;384(24):2273-2282. doi:10.1056/NEJMoa2104983	Unsuitable study design (no comparator group)
30.	Trostle ME, Limaye MA, Avtushka V, Lighter JL, Penfield CA, Roman AS. COVID-19 vaccination in pregnancy: early experience from a single institution. American journal of obstetrics & gynecology MFM. 2021;3(6):100464-100464. doi:10.1016/j.ajogmf.2021.100464	Unsuitable study design (no comparator group)
31.	Zauche LH, Wallace B, Smoots AN, et al. Receipt of mRNA Covid-19 Vaccines and Risk of Spontaneous Abortion. New England Journal of Medicine. 2021;doi:10.1056/NEJMc2113891	Unsuitable study design (no comparator group)

Table S2. Characteristics of the studies included in the systematic review

Authors	Country	Study type	Reported outcome categories	Vaccinated	Unvaccinated	Data collection period	Type of vaccine	Number of doses received	Timing of administration (trimester)
Beharier et al, 2021[19]	Israel (8 Centres across Israel)	Case control	Birthweight in grams<br>Birthweight Z-score*<br>Gestational age at delivery<br>Gestational diabetes<br>HDP<br>Preeclampsia<br>NICU admission<br>Preterm birth below 37 weeks	86	62	For vaccine recipients -January 2021 to March 2021)<br><br>For controls - April 2020 and March 2021	BNT162b2 (Pfizer–BioNTech) m RNA vaccine	Two doses	Not specified<br><br>GA at vaccination (Mean ± SD),weeks – 39.3 ± 1.3<br><br>Maternal age at vaccination (Mean ± SD),years – 31.7 ± 5.8
Blakeway et al, 2021[8]	UK	Cohort	Birthweight Z-score<br>Caesarean section<br>Fetal abnormalities<br>Gestational age at delivery<br>ICU admission<br>Instrumental delivery<br>Intrapartum pyrexia<br>HDU admission<br>NICU admission<br>Placental abruption<br>Postpartum haemorrhage<br>Preterm birth before 37 weeks<br>SGA at birth<br>Stillbirth<br>Suspected chorioamnionitis<br>Vaginal delivery, any<br>Vaginal delivery, unassisted	133	399	March 1st to July 4th 2021	BNT162b2 (Pfizer–BioNTech) m RNA vaccine, Moderna-mRNA-1273 vaccine, ChAdOx1 nCoV-19 viral-vector vaccine (AstraZeneca)	One dose -115<br>Both doses- 26	1st trimester – 0<br>2nd trimester- 20<br>3rd trimester – 121<br><br>GA at vaccination (Mean ± SD),weeks – Not specified<br><br>Maternal age at vaccination, (Median ± IQR)- 35.0 (31.7-37.0)
Bleicher et al, 2021[20]	Israel	Cohort	Composite pregnancy complications<br>COVID-19 positive since last questionnaire	202	124	January 10, 2020 to January 15, 2020	BNT162b2 (Pfizer–BioNTech) m RNA vaccine	One dose – 78 (38.6%)	1st trimester – 36 (17.8%)<br>2nd trimester- 110 (54.5%)

			Fetal growth restriction<br>Pregnancy loss up to 13 weeks' gestation<br>Pregnancy loss 14-28 weeks' gestation<br>Antepartum bleeding<br>Anomaly on anatomy scan<br>HDP<br>Premature contractions<br>Preterm birth					Two doses – 124 (61.4%)	3rd trimester – 56 (27.7%)<br><br>GA at vaccination (Mean ± SD),weeks – 20.14 ± 10.3<br><br>Maternal age at vaccination (Mean ± SD),years – 31.7 ± 3.9
BORNOntario, 2021[21]	Canada	Case control	Stillbirth	31343	76488	January 1, 2021 to October 31, 2021	BNT162b2 (Pfizer–BioNTech) m RNA vaccine, Moderna-mRNA-1273 vaccine, viral-vector vaccine (ChAdOx1 nCoV-19 viral-vector vaccine (AstraZeneca or COVISHIELD)	Not specified	Not specified<br><br>GA at vaccination (Mean ± SD),weeks – Not specified<br><br>Maternal age at vaccination (Mean ± SD),years – Not specified
Bookstein Peretz et al, 2021[13]	Israel	Case-control	Birthweight in grams<br>Caesarean section<br>ELCS<br>EMCS	57	260 non pregnant women	January 2021 to February 2021	BNT162b2 (Pfizer–BioNTech) m RNA vaccine	Two doses – 57 (100%)	3rd Trimester - 57

			Endometritis<br>Gestational age at delivery<br>Gestational diabetes<br>HDP<br>Induction of labour<br>Instrumental delivery<br>Miscarriage<br>Neonatal CPR<br>Neonatal death<br>Neonatal fever<br>Neonatal invasive ventilation<br>Neonatal oxygen<br>NICU admission<br>Oligohydramnios<br>Placental abruption<br>Polyhydramnios<br>Postpartum haemorrhage<br>Preterm birth before 37 weeks' gestation<br>SGA at birth<br>Stillbirth						GA at vaccination (Mean ± SD),weeks – Not specified<br><br>Maternal age at vaccination (Mean ± SD),years – Not specified
Butt et al, 2021[22]	Qatar	Cohort and test-negative case-control	SARS-CoV-2 infection<br>Maternal death	407	407	December 20, 2020 to May 30, 2021	Pfizer-BNT-162b2, Moderna-mRNA-1273 vaccine	2 doses	1st trimester – 323 (79.4%)<br>2nd trimester – 84 (20.6%)<br><br>GA at vaccination (Mean ± SD),weeks – Not specified<br><br>Maternal age at vaccination, (Median ± IQR)- 32 (29–36)

Dagan et al, 2021[23]	Israel (Clalit Health Services)	Cohort	Documented infection with SARS-CoV-2<br>Symptomatic COVID-19<br>COVID-19-related hospitalisation<br>Severe illness and death	10861	10861	December 20 2020 to June 3, 2021	BNT162b2 (Pfizer–BioNTech) m RNA vaccine	Two doses	1st trimester-2814 (26%)<br>2nd trimester-5242 (48%)<br>3rd trimester-2805 (26%)<br><br>GA at vaccination (Mean ± SD),weeks – Not specified<br><br>Maternal age at vaccination (Mean ± SD),years – 30 (26-33)
Goldshtein et al, 2021[24]	Israel (Maccabi Health Care Services, Tel Aviv)	Cohort	Primary outcome: documented SARS-CoV-2 infection28 days or more after the first vaccine dose<br>Other outcomes:<br>Birthweight in grams<br>Gestational age at delivery<br>IUGR<br>Maternal death<br>Miscarriage<br>Pulmonary embolism<br>Preeclampsia<br>Preterm birth before 37 weeks' gestation<br>SARS CoV-2-associated hospitalisation<br>Stillbirth	7530	7530	December 19, 2020-February 28, 2021	BNT162b2 (Pfizer–BioNTech) m RNA vaccine	Not specified	2nd Trimester-46%<br>3rd trimester-33%<br><br>GA at vaccination (Mean ± SD),weeks – Not specified<br><br>Maternal age at vaccination (Mean ± SD),years – 31.1 (5.01)

Gray et al, 2021[25]	USA	Cohort	Assisted ventilation<br>Birthweight in grams<br>Caesarean section<br>Grade 3/4 intraventricular haemorrhage<br>HDP<br>IUGR<br>Necrotising enterocolitis<br>Neonatal sepsis<br>Neonatal death<br>Neonatal oxygen<br>Neonatal seizure<br>NICU admission<br>Preterm birth before 37 weeks' gestation<br>Respiratory distress syndrome<br>Special care nursery admission<br>TTN<br>Vaginal delivery	13	16 non pregnant women	Between December 17, 2020, and February 23, 2021	BNT162b2 (Pfizer–BioNTech) m RNA vaccine, Moderna-mRNA-1273 vaccine	Two doses	3rd trimester (100%)<br><br>GA at vaccination (Mean ± SD),weeks – Not specified<br><br>Maternal age at vaccination (Mean ± SD),years – Not specified for the included cohort of women
Kachikis et al, 2021[26]	COVID-19 Vaccine in Pregnancy and Lactation Registry USA	Cohort	Miscarriage rate following second dose of vaccination	7809 pregnant women,<br>288 delivered	6815 lactating individuals<br><br>2901 non pregnant women	January to March 2021	BNT162b2 (Pfizer–BioNTech) m RNA vaccine,<br>Moderna-mRNA-1273 vaccine,<br>Ad26.COV.2.S (Janssen) vaccine	Two doses-6586	Data available for 7611<br>1st trimester - 1822<br>2nd trimester-3694<br>3rd trimester-2095<br><br>GA at vaccination (Mean ± SD),weeks – Not specified

									Maternal age at vaccination (Mean ± SD),years – Not specified for the included cohort of women
Kharbanda et al, 2021[27]	The Vaccine Safety Datalink USA	Cohort	Spontaneous abortions within 28 days of receipt of COVID-19 vaccine	19824	85622	December 15, 2020 to June 28, 2021	BNT162b2 (Pfizer–BioNTech) m RNA vaccine, Moderna-mRNA-1273 vaccine, Ad26.COV.2.S (Janssen) vaccine	Not specified	Not specified<br><br>GA at vaccination (Mean ± SD),weeks – Not specified<br><br>Maternal age at vaccination (Mean ± SD),years – Not specified for the included cohort of women
Lipkind et al, 2022[28]	Morbidity and Mortality weekly report, CDC, USA	Cohort	Preterm births before 37 weeks' gestation<br>SGA	For preterm birth – 10064<br>For SGA-8928	For preterm birth – 36015<br>For SGA-31699	December 15, 2020 to July 22, 2021	BNT162b2 (Pfizer–BioNTech) m RNA vaccine, Moderna-mRNA-1273 vaccine, Ad26.COV.2.S (Janssen) vaccine	One dose – 1759 (18.2%)<br><br>Two doses-7881 (81.8%)	1st trimester - 172 (1.7%)<br>2nd trimester-3668 (36.5%)<br>3rd trimester-6224 (61.8%)<br><br>GA at vaccination (Mean ± SD),weeks – Not specified

									Maternal age at vaccination (Mean ± SD),years – 32.3 (4.5)
Magnus et al, 2021[29]	Norway	Case-control	Miscarriage within 5 weeks of vaccination<br>Miscarriage within 3 weeks of vaccination	For miscarriage within 5 weeks – 1003<br>For miscarriage within 3 weeks - 595	For miscarriage within 5 weeks – 17474<br>For miscarriage within 3 weeks - 17882	February 15, 2021 to August 15, 2021	BNT162b2 (Pfizer–BioNTech) m RNA vaccine, Moderna-mRNA-1273 vaccine, ChAdOx1 nCoV-19 viral-vector vaccine (AstraZeneca)	One dose -539<br><br>Two doses - 233	Not specified<br><br>GA at vaccination (Mean ± SD),weeks – Not specified<br><br>Maternal age at vaccination (Mean ± SD),years – Not specified
Morgan et al, 2021[30]	USA	Cohort	Severe or critical COVID-19<br>Severe COVID-19<br>Critical COVID-19<br>Positive SARS-COV-2 infection<br>Stillbirth<br>Maternal death<br>ICU admission<br>Remdesivir<br>Dexamethasone<br>Tocilizumab	1332	8760	From June 15, 2021 to August 20, 2021	BNT162b2 (Pfizer–BioNTech) m RNA vaccine 883 (66.3%), Moderna-mRNA-1273 vaccine - 382 (28.7%)<br>Ad26.COV.2.S (Janssen) vaccine - 67 (5.0%)	One dose for JanssenTwo doses for mRNA vaccine)	Not specified<br><br>GA at vaccination (Mean ± SD),weeks – Not specified<br><br>Maternal age at vaccination (Mean ± SD),years – 32.1±5.9
Rottenstreich et al,2021[31]	Israel	Cohort	HDP<br>Diabetes<br>Gestational age at delivery	712	1063	January 19, 2021 to 27 April 2021	BNT162b2 (Pfizer–BioNTech) m RNA vaccine	Two doses	3rd trimester (100%)

			Gestational age at delivery 34 weeks<br>Gestational age at delivery <37 weeks<br>Induction of labour<br>Oxytocin augmentation of labour<br>Epidural analgesia<br>Meconium-stained amniotic fluid<br>Chorioamnionitis<br>Caesarean delivery<br>Elective Caesarean delivery<br>In-labour Caesarean delivery<br>Home/car delivery<br>Vacuum-assisted delivery<br>Hospitalisation length, days<br>Prolonged hospital stay<br>Episiotomy<br>Maternal ICU admission<br>Postpartum haemorrhage<br>Placental abruption<br>Haemoglobin drop, g/dl<br>Haemoglobin drop >4 g/dl<br>Puerperal fever<br>Blood product transfusion<br>Composite adverse maternal outcome<br>Birthweight > 4000 g<br>LGA<br>SGA<br>1-minute Apgar score ≤ 7<br>5-minute Apgar score ≤ 7<br>Intrauterine fetal death<br>NICU admission<br>Meconium aspiration syndrome<br>Jaundice<br>TTN						GA at vaccination (Mean ± SD),weeks – Not specified<br><br>Maternal age at vaccination (Mean ± SD),years – 30.6± 5.8

			Mechanical ventilation<br>Seizures<br>Hypoglycaemia<br>Sepsis<br>Encephalopathy<br>Intracranial haemorrhage<br>Birth asphyxia<br>Composite adverse neonatal outcome						
Theiler et al, 2021[32]	USA<br><br>(Mayo Clinic Health system)	Case-control	3rd or 4th degree laceration<br>5 minute Apgar score <7<br>Adverse Outcomes Index<br>Adverse Outcomes Index excluding laceration<br>Birth trauma<br>Caesarean section<br>Hypoxic Ischaemic Encephalopathy<br>ICU admission<br>Instrumental delivery<br>Low birth weight (<2500g)<br>NICU admit >2500g<br>Pulmonary embolism<br>PIH<br>Postpartum haemorrhage<br>Preeclampsia<br>Preterm birth before 37 weeks' gestation<br>Return to operating theatre<br>Stillbirth<br>Stroke<br>Transfusion<br>Uterine rupture<br>Vaginal delivery, any<br>Vaginal delivery, unassisted<br>Very low birth weight (<1500g)	140	1862	Between December 10, 2020, and April 19, 2021	BNT162b2 (Pfizer–BioNTech) m RNA vaccine<br>-127 women, Moderna-mRNA-1273 vaccine – 12<br>Ad26.COV.2.S (Janssen) vaccine -1	Not specified	Not specified<br><br>GA at vaccination (Median, IQR),weeks – 35.4 (17.2-44.2)<br><br>Maternal age at vaccination (Mean ± SD),years – 31.8 (3.72)

UKHSA 25th Nov 2021 weekly surveillance data[33]	UK	Cohort	Stillbirth rate<br>Low birthweight <2500 g<br>Very low birthweight <1500 g<br>Preterm birth < 37 weeks' gestation<br>Very premature birth < 32 weeks' gestation<br>Extremely premature < 28 weeks' gestation	24,759 (at least one dose)	329158	January 2021, to August 2021	BNT162b2 (Pfizer–BioNTech) m RNA vaccine - 80.8%,<br>ChAdOx1 nCoV-19 viral-vector vaccine (AstraZeneca) - 11.1%,<br>Moderna-mRNA-1273 vaccine - 8.1%	Not specified	Data available for 18,187 women.<br>1st trimester- 695 (3.8%),<br>2nd trimester- 4,487 (24.7%)<br>3rd trimester - 13005(71.5%)<br><br>GA at vaccination (Mean ± SD),weeks – Not specified<br><br>Maternal age at vaccination (Mean ± SD),years – Not specified
Wainstock et al, 2021[34]	Israel	Cohort	Gestational diabetes<br>HDP<br>Oligohydramnios<br>Polyhydramnios<br>Pathological presentation<br>Meconium-stained amniotic fluid<br>Gestational age at delivery<br>Apgar score <7 at 5 minutes<br>Non reassuring fetal monitoring<br>Caesarean delivery<br>Vacuum delivery<br>Placental abruption<br>Postpartum haemorrhage	913	3486	January 2021 to June 2021	BNT162b2 (Pfizer–BioNTech) m RNA vaccine	One dose – 155 (17.0%)<br><br>Two doses – 758 (83.0%)	2nd/3rd trimester (exact numbers not specified)<br><br>GA at vaccination (Mean ± SD),weeks – Not specified<br><br>Maternal age at vaccination (Mean ± SD),years –

			Maternal postpartum fever<br>Length of maternal hospitalisation following Caesarean delivery, days<br>Length of maternal hospitalisation following vaginal delivery, days<br>Birthweight, g<br>SGA<br>Newborn respiratory complications<br>Newborn fever<br>Length of neonatal hospitalisation following Caesarean delivery, days<br>Length of neonatal hospitalisation following vaginal delivery, days						30.6 ± 5.3
Baden et al, 2021[35]	USA	RCT	Miscarriage	6	7	July 27 2020 to October 23, 2020	Moderna-mRNA-1273 vaccine	Two doses	Not specified
Hillson et al, 2021[36]	Brazil<br>South Africa<br>UK	RCT	Miscarriage, excluding Brazilian data<br>Termination, excluding Brazilian data<br>Miscarriage or termination, all<br>Preterm birth	43	24		ChAdOx1 nCoV-19 viral-vector vaccine (AstraZeneca)	Two doses	Not specified
Polack et al, 2020[1]	Argentina<br>Brazil<br>USA	RCT	Miscarriage	11	12	July 27, 2020 to November 14, 2020	BNT162b2 (Pfizer–BioNTech) m RNA vaccine	Two doses	Not specified
Sadoff et al, 2021[37]	Argentina<br>Brazil<br>Chile<br>Columbia<br>Mexico<br>Peru	RCT	Miscarriage	4	4	September 21, 2020 to January 22, 2021	Ad26.COV.2.S (Janssen) vaccine	One dose	Not specified

	South Africa USA								
Voysey et al, 2021[2]	Brazil South Africa UK	RCT	Miscarriage	12	9	April 23 to November 4, 2020	ChAdOx1 nCoV-19 viral-vector vaccine (AstraZeneca)	Two doses	Not specified

GA: Gestational age, SD: Standard deviation, IQR: Inter Quartile range, RCT: randomized controlled trial, HDP: hypertensive disorders of pregnancy, SGA: small for gestational age, PIH: pregnancy induced hypertension, NICU: neonatal intensive care unit, ICU: intensive care unit, TTN: transient tachypnea of newborn, LGA: large for gestational age, IUGR: intrauterine growth restriction, CPR: cardiopulmonary resuscitation, ELCS: elective caesarean section, EMCS: emergency caesarean section

*Estimated using the raw data reported by the authors

Table S3. ROBINS-I risk of bias assessment of observational studies.

Author, year	Confounding	Selection of participants	Classification of interventions	Deviations from interventions	Missing data	Measurement of outcomes	Selection of the reported result	Overall bias
Beharier, 2021	Serious	Moderate	Low	Low	Moderate	Low	Moderate	Serious
Blakeway, 2021	Moderate	Low	Low	Low	Low	Low	Moderate	Moderate
Bleicher 2021	Serious	Serious	Low	Low	Low	Moderate	Low	Serious
BORNOntario, 2021	Moderate	Low	Low	Low	Low	Moderate	Low	Moderate
BooksteinPeretz, 2021	Moderate	Moderate	Low	Low	Low	Moderate	Low	Moderate
Butt, 2021	Moderate	Low	Low	Low	Low	Low	Low	Moderate
Dagan, 2021	Moderate	Low	Low	Low	Low	Low	Low	Moderate
Goldshtein, 2021	Moderate	Low	Low	Low	Low	Low	Moderate	Moderate
Gray, 2021	Moderate	Moderate	Low	Low	Low	Low	Low	Moderate
Kachikis, 2021	Low	Low	Low	Low	Low	Moderate	Low	Moderate
Kharbanda, 2021	Moderate	Low	Low	Low	Low	Low	Low	Moderate
Lipkind, 2022	Moderate	Low	Low	Low	Low	Low	Low	Moderate
Magnus, 2021	Moderate	Low	Low	Low	Low	Low	Low	Moderate
Morgan, 2021	Moderate	Moderate	Low	Low	Low	Low	Low	Moderate
Rottenstreich 2021	Moderate	Low	Low	Low	Low	Low	Low	Moderate
Theiler, 2021	Serious	Low	Low	Low	Low	Low	Moderate	Serious
UKHSA, 2021	Moderate	Low	Low	Low	Low	Moderate	Low	Moderate
Wainstock, 2021	Moderate	Moderate	Low	Low	Low	Low	Low	Moderate

Low risk: comparable to a well-performed randomised trial; moderate risk: sound for a non-randomised study but cannot be compared to a well-performed randomised trial; serious risk: study has some important problems; critical risk: study is too problematic to provide any useful evidence.

Table S4. Cochrane Risk of Bias Tool 2 for randomized trials.

**Author, year**	**Randomisation process**	**Deviations from intended interventions**	**Missing outcome data**	**Measurement of outcome**	**Reported results**	**Overall risk**
Baden, 2021	Low	Low	Low	Low	Low	Low
Voysey, 2020	Low	Low	Low	Low	Low	Low
Polack, 2020	Low	Low	Low	Low	Low	Low
Sadoff, 2021	Low	Low	Low	Low	Low	Low
Hillson, 2021	Low	Low	Low	Low	Low	Low

Table S5. Literature search strategy.

**Set#**	**Search term**
S1	tio,ab(pregnan*)
S2	EMB.EXACT("prenatal care")
S3	MESH.EXACT("Prenatal Care")
S4	EMB.EXACT.EXPLODE("pregnancy") OR EMB.EXACT("pregnant woman")
S5	MESH.EXACT.EXPLODE("Pregnancy") OR MESH.EXACT.EXPLODE("Pregnancy Trimesters") OR MESH.EXACT("Pregnancy Complications") OR MESH.EXACT("Pregnant Women")
S6	(‘coronaviridae’/exp OR coronavirus OR ‘corona virus’) AND (wuhan OR ‘beijing’/exp OR eijing OR shanghai OR ‘2019 ncov’ OR ncovor OR ‘covid 19’ OR ‘sars cov 2’)
S7	(exp coronavirus/ or coronavirus.mp. or corona virus) and (wuhan or eijing or shanghai or 2019-nCoV or nCovor or COVID-19 or SARS-CoV-2)
S8	EMB.EXACT.EXPLODE(“CORONAVIRIDAE INFECTION”) OR EMB.EXACT.EXPLODE(“CORONAVIRUS INFECTIONS”)
S9	tio,ab(“COVID-19” or “SARS-CoV-2” or “2019-nCoV” or “n-CoV” and “coronavirus”)
S10	tio,ab(Immuniz* OR immunis* OR vaccin*)
S11	S5 OR S4 OR S3 OR S2 OR S1
S12	S9 OR S8 OR S7 OR S6
S13	S12 AND S11 AND S10
S14	(S12 AND S11 AND S10) and (pd(2020-2021))
S15	(S12 AND S11 AND S10) and (pd(20201101-20211201))
S16	(S12 AND S11 AND S10) and (pd(20201101-20211201) and (pd(20201201-20210830)))
